# Evaluation of biological safety in vitro and immunogenicity in vivo of recombinant *Escherichia coli* Shiga toxoids as candidate vaccines in cattle

**DOI:** 10.1186/s13567-015-0175-2

**Published:** 2015-04-10

**Authors:** Katharina Kerner, Philip S Bridger, Gabriele Köpf, Julia Fröhlich, Stefanie Barth, Hermann Willems, Rolf Bauerfeind, Georg Baljer, Christian Menge

**Affiliations:** Institute of Hygiene and Infectious Diseases of Animals, Justus Liebig University, Frankfurter Str. 85-89, 35392 Giessen, Germany; Current Address: Friedrich-Loeffler-Institut, Institute of Molecular Pathogenesis, Naumburger Str. 96a, 07743 Jena, Germany; Clinic for Ruminants and Swine (Internal Medicine & Surgery), Justus Liebig University, Giessen, Germany

## Abstract

Cattle are the most important reservoir for enterohemorrhagic *Escherichia coli* (EHEC), a subset of shigatoxigenic *E. coli* (STEC) capable of causing life-threatening infectious diseases in humans. In cattle, Shiga toxins (Stx) suppress the immune system thereby promoting long-term STEC shedding. First infections of animals at calves’ age coincide with the lack of Stx-specific antibodies. We hypothesize that vaccination of calves against Shiga toxins prior to STEC infection may help to prevent the establishment of a persistent type of infection. The objectives of this study were to generate recombinant Shiga toxoids (rStx1_mut_ & rStx2_mut_) by site-directed mutagenesis and to assess their immunomodulatory, antigenic, and immunogenic properties. Cultures of bovine primary immune cells were used as test systems. In ileal intraepithelial lymphocytes both, recombinant wild type Stx1 (rStx1_WT_) and rStx2_WT_ significantly induced transcription of IL-4 mRNA. rStx1_WT_ and rStx2_WT_ reduced the expression of Stx-receptor CD77 (syn. Globotriaosylceramide, Gb3) on B and T cells from peripheral blood and of CD14 on monocyte-derived macrophages. At the same concentrations, rStx1_mut_ and rStx2_mut_ exhibited neither of these effects. Antibodies in sera of cattle naturally infected with STEC recognized the rStx_mut_ toxoids equally well as the recombinant wild type toxins. Immunization of calves with rStx1_mut_ plus rStx2_mut_ led to induction of antibodies neutralizing Stx1 and Stx2. While keeping their antigenicity and immunogenicity recombinant Shiga toxoids are devoid of the immunosuppressive properties of the corresponding wild type toxins in cattle and candidate vaccines to mitigate long-term STEC shedding by the reservoir host.

## Introduction

Enterohemorrhagic *Escherichia coli* (EHEC), a subset of Shiga toxin-producing *E. coli* (STEC), are food-borne pathogens which can evoke life-threatening diseases, such as hemorrhagic colitis and hemolytic-uremic syndrome, in humans. Cattle and other ruminants are primary reservoirs for EHEC serotypes that are frequently associated with human disease, e.g., EHEC O157:H7. Calves become infected with a plethora of different STEC strains early in life via horizontal or vertical transmission. Although calves rarely develop clinical signs of STEC infection they may shed these bacteria for several months and shed STEC quantities may be considerably high at some sampling points [1-4].

To prevent humans from EHEC infection, interventions must be applied at several stages of the food chain, starting in the animal itself and continuing in slaughterhouses, processing plants, distributors, and households [5]. A systematic review of vaccinations to reduce the shedding of *E. coli* O157 in the faeces of domestic ruminants revealed that vaccination may be a sensible control option [6]. Current vaccination strategies are promising but only succeed partially in reducing *E. coli* O157:H7 excretion (as reviewed by [5]). In some instances, e.g., when vaccinating cattle against H7 flagellin, an important adhesion factor to bovine intestinal epithelium during early stages of colonization [7], systemically induced H7-specific IgG may even impair innate immune responses to *E. coli* O157:H7 when getting into contact with the epithelium via neutralisation of TLR5-mediated activation of epithelial cells [5].

Shiga toxins (Stx) are potent protein cytotoxins and represent the principal STEC virulence factor in the pathogenesis of human infections. Cumulating evidence exist that Stx act as immunomodulating agents during STEC infections in cattle. Stx1 alters the cytokine expression pattern in mucosal macrophages [8] and intraepithelial lymphocytes [9] and suppresses the activation and proliferation of mucosal [10] and peripheral lymphocytes in vitro [11]. The development of an adaptive cellular immune response is significantly delayed following experimental infection of calves with Stx2^+^ STEC O157:H7 compared to that in animals inoculated with Stx-negative *E. coli* O157:H7 [12]. In vitro and in vivo studies revealed that Stx operate during the early phases of immune activation rather than depressing an established immunity [11-14]. Consequently, Stx likely acts as immunomodulator only upon first STEC infection of hitherto immunologically naïve calves. Of note, a significant portion of calves lacks anti-Stx antibodies at the time of first encountering STEC [2]. We hypothesize that passive (maternal) and active vaccination against Stx1 and Stx2 confers a protection against the toxins’ immunosuppressive effects and subsequently enables the calves to actively mount a rapid immune response against STEC strains circulating in the respective cohort. Kuribayashi et al. showed that immunization of pregnant cows with Stxs led to an enrichment of colostra with anti-Stx1 and anti-Stx2 antibodies [15]. Subsequent application of bovine colostral anti-Stx2 to experimentally infected dogs indeed reduced STEC shedding [16].

Development of anti-Stx antibodies is remarkably delayed after natural [2] and experimental STEC infection of cattle [17]. Although Stx primarily targets CD8^+^ cells [11], the immunomodulating capacity of Stx may also impair the humoral anti-Stx response. A strategy to circumvent this obstacle is the use of toxoid vaccines. Chemically inactivated Stx2e, however, was only partially effective in protecting piglets against oedema disease [18]. A more promising approach is the inactivation of Stx by genetic modification. Replacement of amino acids E167 and R170, located within the enzymatically active cleft of Stx2e [19,20] and vaccination of piglets with the recombinant protein fully protected piglets during challenge with native Stx2e [21]. Similar results have been reported for mice [22,23].

In order to follow a novel approach to add on or to improve current vaccination strategies to mitigate STEC shedding by cattle, the objectives of this proof-of-concept study were to generate recombinant Shiga toxoids (rStx1_mut_ & rStx2_mut_) by site-directed mutagenesis and to assess the immunomodulatory, antigenic, and immunogenic properties of the resulting proteins in cattle.

## Material and methods

### Generation of recombinant toxins and toxoids for in vitro and in vivo applications

For generating recombinant Stx (rStx_WT_) and Stx mutants (rStx_mut_), *stx1* and *stx2* genes from the *E. coli* reference strain EDL 933 (ATCC 43895) were PCR amplified (primers [5′ → 3′], Stx1_for: GGAGTATTGTGTCATATGAAAAT, Stx1_rev: TATTCGAATTCAACGAAAAATAA, Stx2_for: TATATGCATATGAAGTGTATATTATTTAAA, Stx2_rev: AACCGTGAATTCAGTCATTATTAAACTGCACT). After restriction of PCR products with *Nde*I and *Eco*RI, resulting fragments were ligated into a compatible pET-24(b) + plasmid vector (Novagen, Merck KGaA, Darmstadt, Germany). Recombinant plasmids were transformed into *E. coli* BLR(DE3) and plasmid DNA bearing the *stx1* and *stx2* inserts, respectively, was prepared for site-directed mutagenesis. To replace E167 and R170 with glutamine (Q) and leucine (L), respectively, we used the QuickChange® Site-Directed Mutagenesis Kit (Stratagene, Amsterdam, The Netherlands). Sequencing of recombinant plasmids revealed, that the gene sequences of the wild type toxins rStx1_WT_ and rStx2_WT_ were identical with the original sequences (Acc.No. AE005174 for Stx1, NC_000924 for Stx2) and those of the mutant toxins rStx1_mut_ and rStx2_mut_ contained the desired mutations (E167Q, R170L; Table 1). Both, rStx_WT_ and rStx_mut_ were expressed in *E. coli* BLR(DE3). Control preparations were obtained from *E. coli* BLR(DE3) transformed with an empty vector (vector control). After incubation of the bacterial pellet with Polymyxin B (1 mg/mL) expressed toxin was collected from the periplasmic space and depleted from endotoxin (Detoxi-Gel™ Endotoxin Removing Gel, Thermo Scientific, Nidderau, Germany).

**Table 1:** **Comparison of the gene sequences of Stx1 and Stx2 before and after mutagenesis**

**Gene**	Relevant nucleotide sequence (codon triplets for amino acids 164 to 174 in 5′ to 3′ direction)*	**Compared to wild type amino acid replacements on position** ^**†**^
*stx1*_*WT*_	-gtg aca gct **g**aa gct tta c**g**t ttt cgg caa ata-	none
*stx1*_*mut*_	-gtg aca gct **C**aa gct tta c**T**t ttt cgg caa ata-	E167Q, R170L
*stx2*_*WT*_	-gtc aca gca **g**aa gcc tta c**g**c ttc agg cag ata-	none
*stx2*_*mut*_	-gtc aca gca **C**aa gcc tta c**T**c ttc agg cag ata-	E167Q, R170L

*Bold letters indicate positions of replaced nucleotides, with small letters marking the nucleotides in wild type toxin sequences and capital letters marking replaced nucleotides in mutant toxin sequences.

^**†**^E = glutamine acid, Q = glutamine; R = arginine; L = leucine.

Quantification of rStx_WT_ was done by VCA and Stx ELISA (see below), quantification of rStx_mut_ only by Stx ELISA. For adjustment of the vector control, the lowest dilution determined for rStx_WT_/rStx_mut_ preparations to be applied in functional assays was also used for the vector control. The content of endotoxin was 51 fg/mL or less in rStx_WT_, rStx_mut_, and vector control preparations at working dilutions.

### Vero cell cytotoxicity assay (VCA) and Vero cell cytotoxicity neutralization assay (VNA)

The VCA was performed in 96-well microtiter plates (Nunc, Wiesbaden, Germany) using Vero cells (ATCC CRL 1587, LGC-Promochem GmbH, Wesel, Germany) as previously described [24] to determine the cytotoxicity (verocytotoxic doses 50%, CD_50_/mL) of the rStx_WT_ preparations and for adjustment of stock solutions (20 000 CD_50_/mL).

The VNA was used for the determination of the neutralization activity in serum of vaccinated calves and was done as previously described [2] in order to determine the titre of neutralizing antibodies [nAb titre] against either wild type Stx1 or wild type Stx2 (Sigma-Aldrich Chemie GmbH, Taufkirchen, Germany).

### Enzyme-linked immunosorbent assay (ELISA)

To quantify rStx protein in the preparations, a commercial Stx ELISA was used (Novitec® Verotoxin ELISA-Test, HISS Diagnostics, Freiburg, Germany) following the manufactures instructions. rStx_mut_ concentrations used for the functional assays were adjusted to reach an OD equivalent to the OD of rStx_WT_ stock solutions containing 20 000 CD_50_/mL. Stock solutions were further diluted accordingly to reach a final concentration of 200 CD_50_/mL or equivalent doses.

Stx-specific antibodies in sera from naturally exposed calves collected during a proceeding study [2] were analysed in a modified form of the ELISA. Briefly, 19 serum samples with Stx1 nAb titres between 60 and 2000 (as determined by VNA) were pre-diluted to achieve an approx. 50% reduction of the relative optical density [OD_rel_]. Four serum samples with a Stx2 nAb titre of 30 were used un-diluted. Serum samples were incubated with either rStx_WT_ or rStx_mut_ preparations for 30 min at 37 °C. Subsequently, pre-incubated rStx was used as sample in the ELISA assay and subsequent steps were performed as described by the manufacturer. OD_rel_ was calculated by the following formula: OD_rel_ [%] = (OD_rStx + serum sample_ - OD_vector control + serum sample_)/(OD_rStx + negative serum_ - OD_vector control + serum sample_) × 100.

### Primary cell cultures

Peripheral blood mononuclear cells (PBMC) were isolated as previously described [11]. Cells were diluted in cell culture medium 1 (RPMI 1640, 10% fetal calf sera, 1% Penicillin/Streptomycin, 0.03% 1 mM 2-β-mercaptoethanol) to 1.5 × 10^6^/mL and aliquoted into a 96-well flat-bottom plate (Greiner Bio-One, Frickenhausen, Germany) at 150 μL per well (2.25 × 10^5^/well). Challenge material (rStx_WT_, rStx_mut_ or vector control, respectively) was added to reach a final concentration of 200 CD_50_/mL or equivalent doses, respectively. For proliferation the mitogen phytohemagglutinin P [PHA-P] was added in a final concentration of 5 μg/mL. Plates were incubated for 96 h in 5% CO_2_ at 37 °C.

Ileal intraepithelial lymphocytes (iIEL) were isolated as described elsewhere [25]. Cells were harvested and diluted in cell culture medium 2 (RPMI 1640, 20% fetal calf sera, 1% Penicillin/Streptomycin, 1% Amphotericin B, 0.01% Gentamicin) to 2 × 10^7^/mL. Nine millilitre of this cell suspension were pipetted into a well of a 6-well plate and challenge material (rStx_WT_, rStx_mut_ or vector control) was added to a final concentration of 200 CD_50_/mL or equivalent doses. Additionally, PHA-P was added to a final concentration of 2.5 μg/mL. Plates were incubated for 6 h in 5% CO_2_ at 37 °C.

Monocyte-derived macrophages (MDM) were isolated as described elsewhere [26,27]. Briefly, a whole blood sample was centrifuged (2380 × *g*, 20 min) and the buffy coat was collected. After several washing and lysis steps, buffy coat was layered onto Ficoll for density centrifugation (800 × *g*, 45 min). Cells were collected by taking the interphase and washed three times with PBS buffer. Cells were adjusted to 4 × 10^6^/mL in cell culture medium 3 (Iscove’s Modified Dulbecco’s Medium (IMDM) without Phenol Red, 20% fetal calf sera, 1% Penicillin/Streptomycin, 1% Amphotericin B, 0.05% 100 mM 2-β-mercaptoethanol) and 25 mL of this cell suspension were transferred to Teflon bags (VueLife Bags, American Fluoroseal Corp., Gaithersburg, USA) and incubated for 8 days (37 °C, 5% CO_2_). At the end of the incubation period, cells were harvested and diluted to 2 × 10^6^/mL in cell culture medium 4 (IMDM without phenol red, 2% fetal calf sera, 1% Penicillin/Streptomycin, 1% Amphotericin B, 0.05% 100 mM 2-β-mercaptoethanol). Five millilitre of the cell suspension was cultured in petri dishes (Greiner Bio-One, Frickenhausen, Germany) for 18 h. Lymphocytes were removed by careful washing and adherent MDMs were left within the dishes in cell culture medium 4. Challenge material was added in cell culture medium 4 to reach a final concentration of 200 CD_50_/mL or equivalent doses and incubated for 6 h.

### Immunophenotyping

At the end of the respective incubation times (see above), cells were resuspended and transferred to V-shape microtitre plates (Greiner Bio-One, Frickenhausen, Germany). After centrifugation (300 × *g*, 3 min, 4 °C) supernatants were flicked out. Pellets were resuspended in washing buffer (PBS supplemented with 1% bovine serum albumin, 0.01% sodium azide, and 0.5% goat serum) as negative control or with 50 μL of primary antibody dilution (diluted 1:50 through 1:500 in washing buffer). Antibodies were purchased from VMRD (Labor Diagnostik Leipzig, Leipzig, Germany; CD4 clone IL-A11, CD8β clone BAT82A, CD14 clone CAM36A, CD21 clone GB25A, γδT/N24 clone GB21A), AbDSerotech (Puchheim, Germany; CD77 clone 38–13) or kindly provided by Dirk Werling (The Royal Veterinary College, London, United Kingdom; CD80 clone N32/52-3, CD86 clone IL-A190). Cells were incubated for 20 min on ice, washed with washing buffer and resuspended in 50 μL of washing buffer with secondary antibodies (fluorescein isothiocyanate (FITC)-labelled α-rat IgM (Dianova GmbH, Hamburg, Germany); allophycocyanin (APC)-labelled α-mouse IgG_1_, APC-labelled α-mouse IgG_2a_, APC-labelled α-mouse IgG_2b_ (Jackson ImmunoResearch Europe Ltd., Suffolk, United Kingdom)) supplemented with 7-amino actinomycin D (7-AAD; final concentration 2 μg/mL; Sigma-Aldrich, Taufkirchen, Germany). After 20 min on ice, cells were washed with washing buffer and analysed with BD FACSCalibur™ Analyzer (Becton-Dickinson, Heidelberg, Germany). For analysis of PBMC, following the last incubation step, cells were incubated with 50 μL of Annexin V-phycoerythrin[PE]-Dilution (1:500; Dianova GmbH, Hamburg, Germany), washed with Annexin V binding buffer (10 mM HEPES pH 7.4, 140 mM NaCl, 2.5 mM CaCl_2_), diluted in Annexin V binding buffer and analysed. Cells were gated according to their size and granularity. Only morphologically intact cells were used for further analysis. Cells positive for 7-AAD uptake or Annexin-V-PE binding were excluded and defined as early apoptotic (positive for Annexin-V-PE), late apoptotic (positive for Annexin-V-PE and 7-AAD), and necrotic (positive for 7-AAD), respectively. Data analysis was performed with FCSExpress (Version 2, De Novo-Software, Thornhill, Ontario, Canada).

### RNA isolation

At the end of the incubation period, iIEL were resuspended, transferred to 50 mL tubes, washed with PBS (200 × *g*, 7 min), lysed in 600 μL RLT buffer (RNeasy MiniKit, Qiagen, Hilden, Germany) supplemented with 1% β-mercaptoethanol, and stored at −70 °C.

All samples were thawed at 37 °C for 5 min, and then homogenized by passing through a 20 G needle. RNA isolation was performed with the RNeasy MiniKit following the manufacturers’ instruction with modifications described by Moussay et al. [9]. Reverse transcription and real-time PCR using primers and probes labelled at the 5′-end with the reporter dye FAM (6-carboxyfluorescein) and at the 3′-end with the quencher dye TAMRA (6-carboxytetramethyl-rhodamine) was conducted as described [9]. PCR amplification was performed on an automated fluorometer (ABI PRISM™ 5700 Sequence Detection System, Applied Biosystems) using 96-well optical plates. Each sample was analysed in duplicates. For analysis of the data, the comparative C_t_ method (ΔΔC_t_ method) was applied with first, normalization of the C_t_ values referring to the housekeeping gene GAPDH and second, comparing the C_t_ values for the quantitation of IL-4-specific mRNA in cultures treated with challenge material (rStx_WT_, rStx_mut_) and in cultures treated with vector control (control cultures) [9].

### Immunization study

The experiment was carried out in strict accordance with European and German laws for the care and use of animals, approved by Thüringer Landesamt für Lebensmittelsicherheit und Verbraucherschutz, Bad Langensalza, Germany (permit no. 22-268-04-04-105/11).

Prior to the experiment, two conventionally raised bull calves aged 11 months tested negative for Stx-specific antibodies (16 and 4 weeks before the trial by VNA). Calves were tested for STEC shedding 16 weeks before the trial and immediately prior to the 1st and the 2nd vaccination. For this purpose coliform bacteria from fecal samples were enriched by growth on Gassner agar (3 plates per sample) [28]. Subsequently, the enriched bacterial culture material was tested with a *stx1*/*stx2*-duplex-PCR modified from Nguyen et al. [29]. While all fecal samples were *stx*-negative at the first and last sampling, in both calves genes encoding Stx1 and Stx2 were present in the fecal sample taken prior to the 1st vaccination at low frequencies (only 1 of 3 enrichment cultures from these fecal samples tested positive). Calves were double-vaccinated i.m. with both, a rStx1_mut_ vaccine and a rStx2_mut_ vaccine on trial days 0 and 21. Immediately prior to application, vaccines had been freshly prepared as follows: rStx1_mut_ and rStx2_mut_ preparations were diluted separately with NaCl solution (0.89%) to 1 000 000 CD_50_ equivalents in 1.4 mL and then supplemented with 0.6 mL of aluminium hydroxide (Alu-Gel-S, Serva Electrophoresis GmbH, Heidelberg, Germany). Blood samples were taken weekly, centrifuged, and sera were frozen at −20 °C. Nine weeks after first immunization, last samples were drawn. Detection of specific antibodies in the sera was done by VNA. Titres below the detection limit were given an arbitrary value of 30.

### Statistical analysis

Unless otherwise indicated, data obtained after applying rStx_WT_ and rStx_mut_ preparations in biological assays were normalized relative to data obtained after application of vector control.

Statistical analysis was done with “SPSS for windows” (Version 15, SPSS Inc., Chicago, Illinois, USA). Single factor variance analyses with repeated measurements were carried out applying Greenhouse Geisser Test for all data from in vitro testing of the preparations. Pearsons’s correlation analysis was used to compare quantitative values from the VNA. Two-tailed p-values with *p* ≤ 0.05 were considered significant. The following description was used: n.s. = not significant (*p* > 0.05); * = *p* ≤ 0.05; ** = *p* ≤ 0.01; *** = *p* ≤ 0.001.

## Results

### Generation of Shiga toxins and toxoids

**Figure 1:**
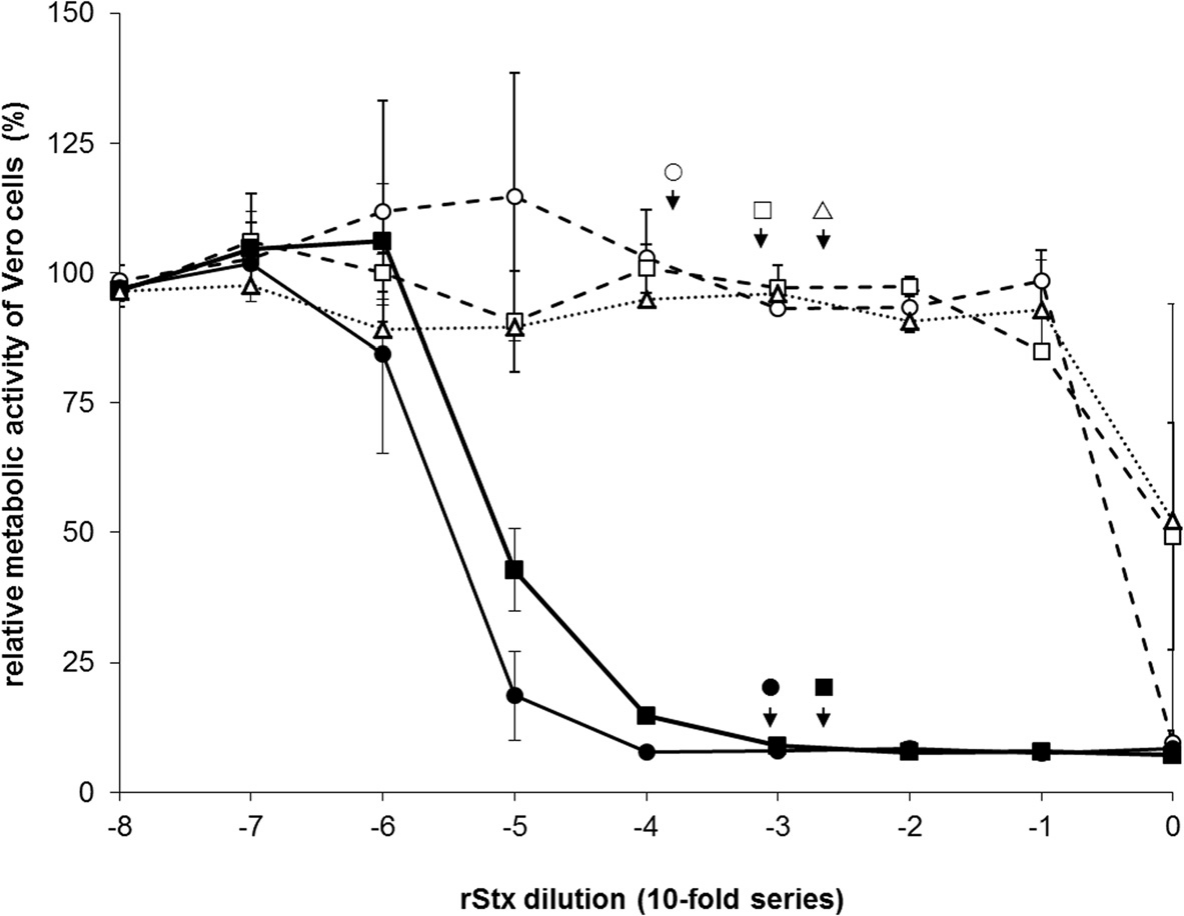
**Effect of recombinant Shiga toxins and toxoids on the cellular metabolic activity of Vero cells.** Cells were incubated for 96 h at 37 °C with 10-fold dilutions of endotoxin-deprived lysates prepared from *E. coli* BLR(DE3) transformed with plasmids encoding for rStx1_WT_ (filled circle, solid line), rStx1_mut_ (open circle, dashed line), rStx2_WT_ (filled square, solid line), rStx2_mut_ (open square, dashed line) or vector control (open triangle, dashed line). Results of VCA are presented relative to data obtained with cells incubated with plain medium as negative control (set to 100%) and data from cells treated with 1% SDS as positive control (set to 0%). Data is depicted as means ± standard deviations from duplicate determinations in one representative out of four independent experiments. Missing error bars are within symbols. For functional assays with bovine primary cell cultures, lysates containing rStx_WT_ were adjusted to reach a final concentration of 200 verocytotoxic doses 50% per mL. Lysates containing rStx_mut_ were diluted to yield the same OD as the corresponding rStx_WT_–containing lysate in an ELISA assay (for details see Material and methods). To visualize the verocytotoxic activities of the respective rStx working dilutions, the calculated dilution factors are depicted by arrows and a corresponding symbol in the diagram.

Lysates from *E. coli* BLR(DE3) transformed with plasmids coding for either of the rStx_WT_ possessed a considerable Vero cytotoxicity (2.7 × 10^6^ and 0.8 × 10^6^ CD_50_/mL for rStx1_WT_ and rStx2_WT_, respectively; geometric mean of *n* = 4 determinations; Figure 1). Lysates containing rStx_mut_ only had low cytotoxic activities (40 and < 20 CD_50_/mL for rStx1_mut_ and rStx2_mut_, respectively) not different from lysates of *E. coli* BLR(DE3) transformed with the empty expression vector (<20 CD_50_/mL). In order to functionally test rStx_WT_ and rStx_mut_ at comparable yet biologically relevant concentrations, a concentration of 200 CD_50_/mL was chosen and rStx_mut_ containing lysates were adjusted to their rStx_WT_ containing counterparts according to the results of an ELISA test (resulting working dilutions indicated in Figure 1).

### Viability and phenotype of bovine PBMC upon in vitro challenge with rStx_WT_ and rStx_mut_

Purified wild type Stx1 from *E. coli* blocks activation and proliferation of bovine lymphocyte subpopulations in vitro without inducing cellular death [11]. Neither incubation of PHA-P (phytohemagglutinin-P) stimulated PBMC with rStx_WT_ nor incubation with rStx_mut_ led to a significant increase in the percentage of late apoptotic/necrotic cells and the percentage of early apoptotic cells as compared to PHA-P stimulated bovine PBMC cultures incubated in the presence of the vector control (referred to as “control cultures” throughout; data not shown).

**Figure 2:**
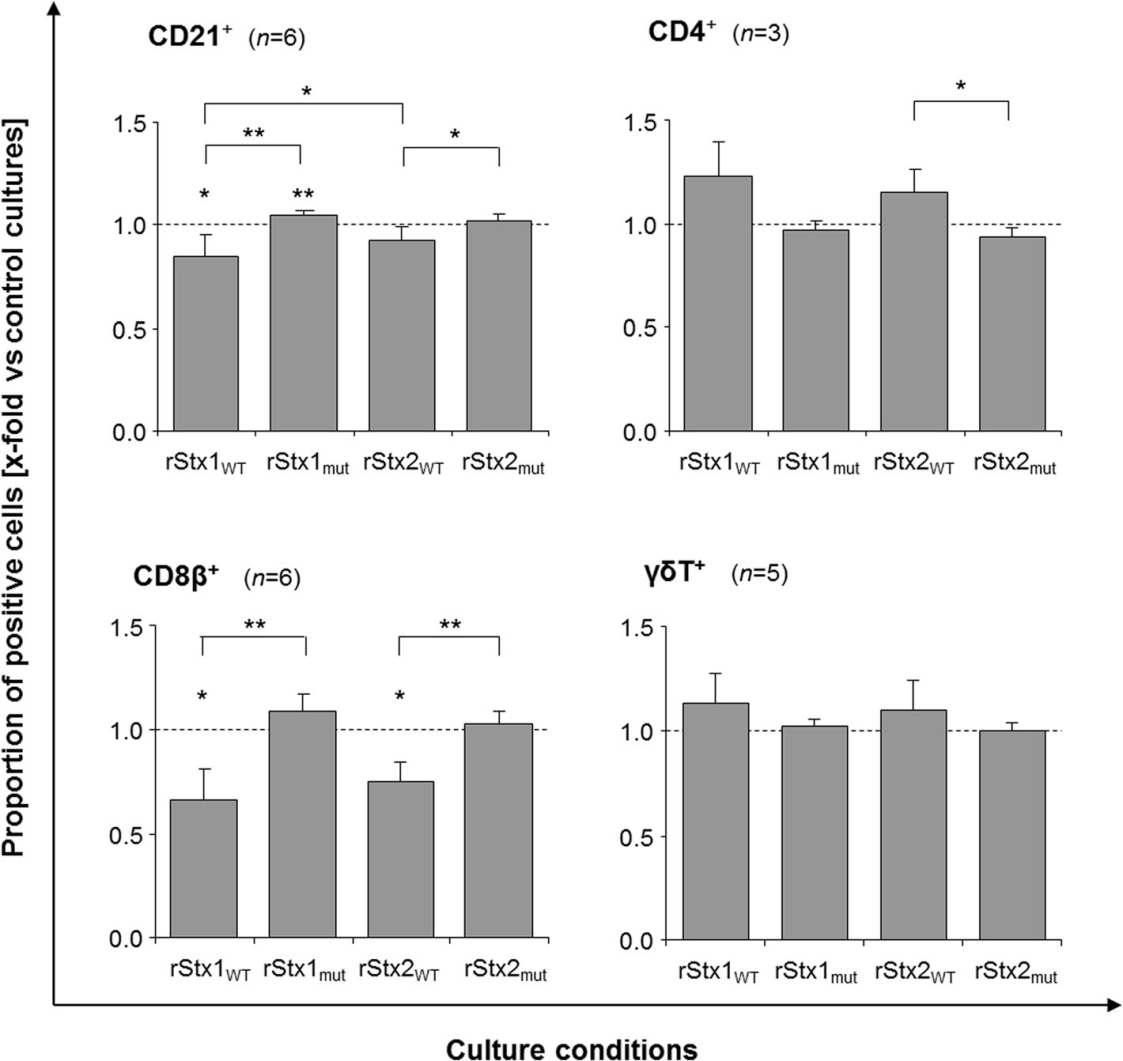
**Proportion of CD21** ^**+**^ **, CD4** ^**+**^ **, CD8β** ^**+**^ **, and** **γδT** ^**+**^ **cells in cultures of PHA-P stimulated bovine PBMC after incubation with recombinant Shiga toxins and toxoids.** Results are shown relative to data obtained from cultures incubated in the presence of the vector control (control cultures; defined as 1.0, indicated by the dashed line). Data is depicted as means ± standard deviations of 3 to 6 repetitive experiments as indicated. ANOVA was performed (1) comparing non-normalized data with the values from control cultures (asterisks above bars) and (2) comparing values of normalized data obtained after incubation with rStx1_WT_ versus rStx1_mut_, rStx2_WT_ versus rStx2_mut_, rStx1_WT_ versus rStx2_WT_, and rStx1_mut_ versus rStx2_mut_ (asterisks above brackets). Significance levels were defined as *p* ≤ 0.001 [***], *p* ≤ 0.01 [**], and *p* ≤ 0.05 [*].

Control cultures phenotyped after four days of in vitro maintenance consisted of 13.2 ± 6.4%, 9.8 ± 4.9%, 30.0 ± 8.4%, and 18.9 ± 6.0% (mean ± standard deviation; *n* = 3–6) of CD4^+^, CD8β^+^, γδT^+^, and B cells (CD21^+^), respectively (data not shown). Addition of rStx1_WT_ or rStx2_WT_ preparations to the culture medium both significantly reduced the portion of CD8β^+^ PBMC while incubation with rStx_mut_ left the proportion of CD8β^+^ cells unaffected (Figure 2). Similarly, incubation with rStx1_WT_ and rStx2_WT_ reduced the portion of CD21^+^ PBMC in the cultures compared to rStx_mut_ treated cells. In turn, the portion of CD4^+^ cells increased after incubation with rStx_WT_ (significant for rStx2_WT_ only). Incubation with rStx_mut_ did not result in significant changes in PBMC composition except a slight but significant increase of CD21^+^ PBMC after challenge with rStx1_mut_ (Figure 2).

**Figure 3:**
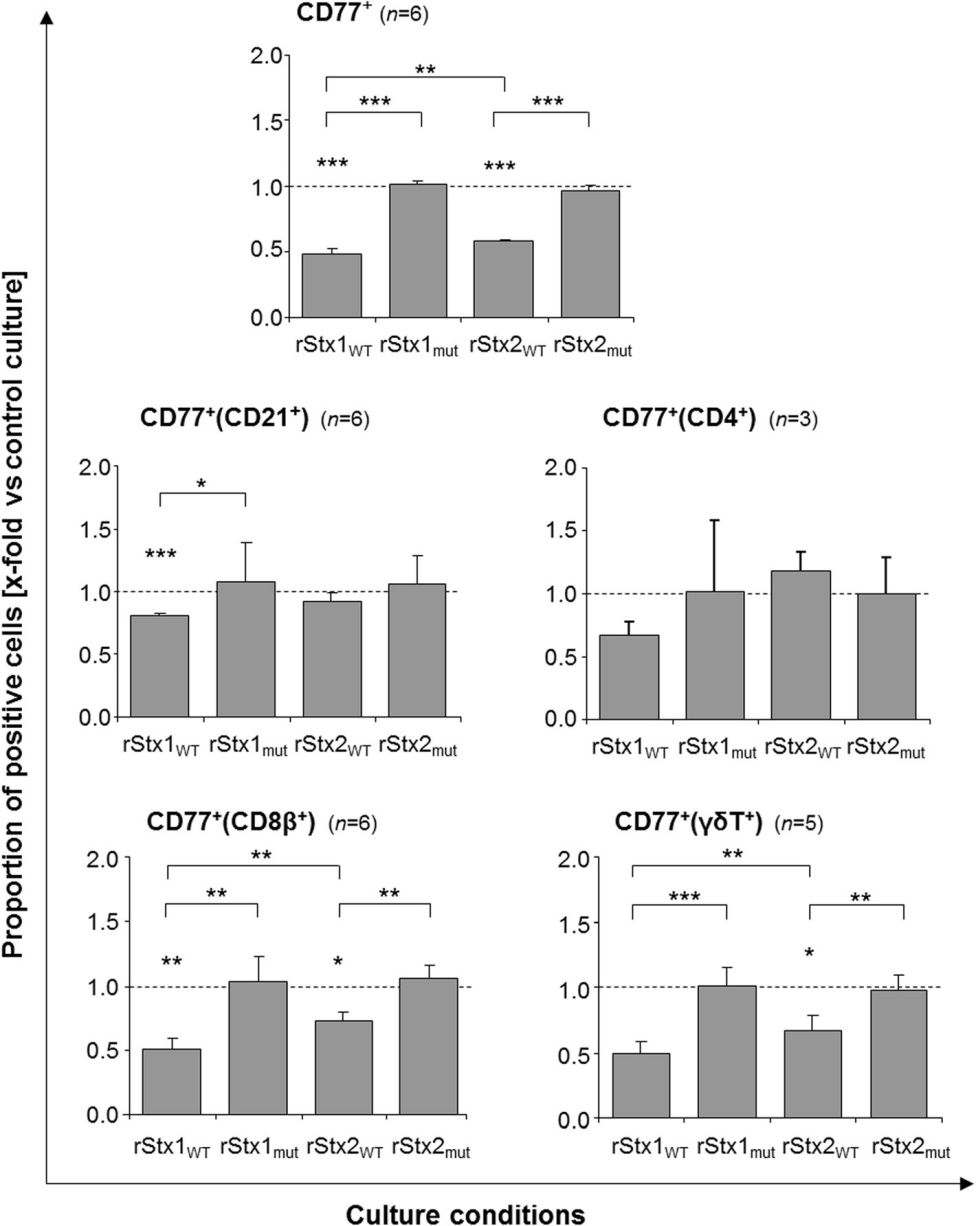
**Bovine PBMC subsets co-expressing CD77 after in vitro challenge with recombinant Shiga toxins and toxoids.** Proportions of bovine PBMC and of PBMC subsets co-expressing CD77 in PHA-P stimulated cultures are shown relative to data obtained from PHA-P stimulated cultures incubated in the presence of the vector control (control cultures; defined as 1.0, indicated by the dashed line). Data is depicted as means ± standard deviations of 3 to 6 repetitive experiments as indicated. Statistical analysis was performed as described in legend to Figure 2. Significance levels were defined as *p* ≤ 0.001 [***], *p* ≤ 0.01 [**], and *p* ≤ 0.05 [*].

**Figure 4:**
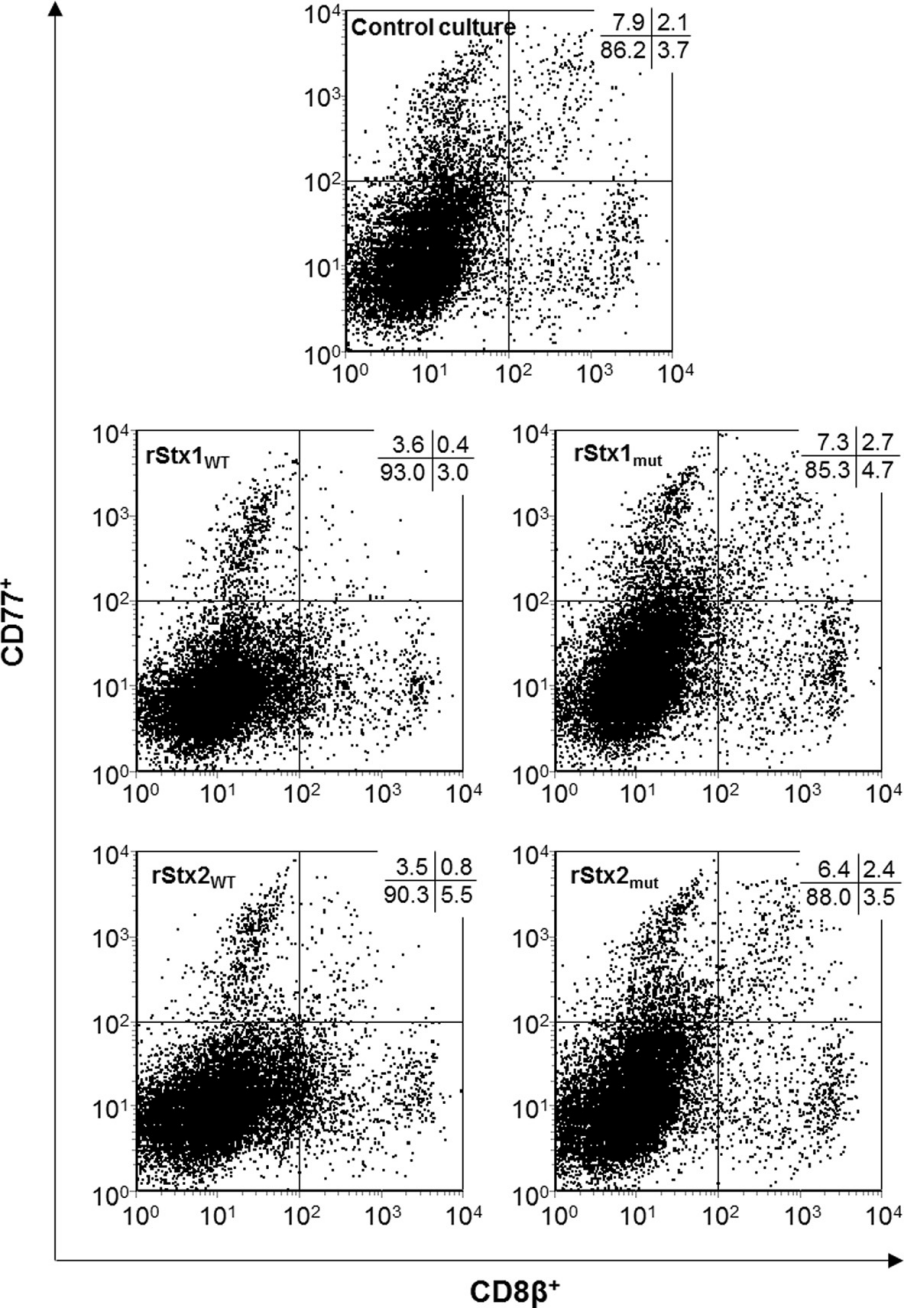
**Effect of recombinant Shiga toxins and toxoids on CD77 expression by bovine CD8β** ^**+**^ **PBMC.** Cells were incubated in culture medium containing 5 μg/mL PHA-P and rStx_WT_ or rStx_mut_ as indicated. After four days of incubation cells were submitted to immunolabelling and flow cytometry analysis. Percentages of CD8β^+^ PBMC co-expressing CD77 (events in the upper right quadrant) are given in the upper right corner of the dot plots. PBMC incubated in the presence of PHA-P and vector control were used as control (upper graph).

CD77 acts as the Stx receptor on a variety of cells from different species including bovine lymphocytes [14] and is up-regulated by bovine lymphocytes upon activation in vitro and in vivo [13]. Sustained down-regulation of CD77 by bovine PBMC is a hallmark of the activity purified wild type Stx1 exerts in bovine PBMC cultures [30]. Incubation with rStx1_WT_ and rStx2_WT_ also caused a significant reduction of the percentage of PBMC expressing CD77 to about half the values detected in control cultures (Figure 3) while incubation with rStx1_mut_ and rStx2_mut_ had no effect. In control cultures, 10.7 ± 1.4% (mean ± standard deviation; *n* = 6) of all PBMC expressed CD77 but the portion of CD77-expressing cells varied between the PBMC subsets analysed. Four days after initiation of cultures, 12.3 ± 9.6%, 23.5 ± 11.5%, 22.9 ± 12.6%, and 29.4 ± 9.6% of CD4^+^, CD8β^+^, γδT^+^, and B cells (CD21^+^), respectively, co-expressed CD77. Effects of rStx1_WT_ and rStx2_WT_ on CD77^+^ cells differed between subsets and showed no correlation with the percentage of cells co-expressing CD77 in that subset. While a comparably high proportion of CD8β^+^ cells (Figure 4) and γδT cells co-expressed CD77 and the portion of CD77^+^ cells was significantly reduced by exposure to rStx_WT_ (Figures 3 and 4), toxins exhibited minor effects on CD21^+^ B cells also expressing CD77 in high numbers in control cultures (Figure 3). CD4^+^ cells showed little CD77 expression which was clearly albeit not significantly reduced in the presence of rStx1_WT_ only (analysis of variance [ANOVA], *p* = 0.378). Incubation with rStx1_mut_ and rStx2_mut_ did not reduce CD77 expression by any of the PBMC subsets.

**Figure 5:**
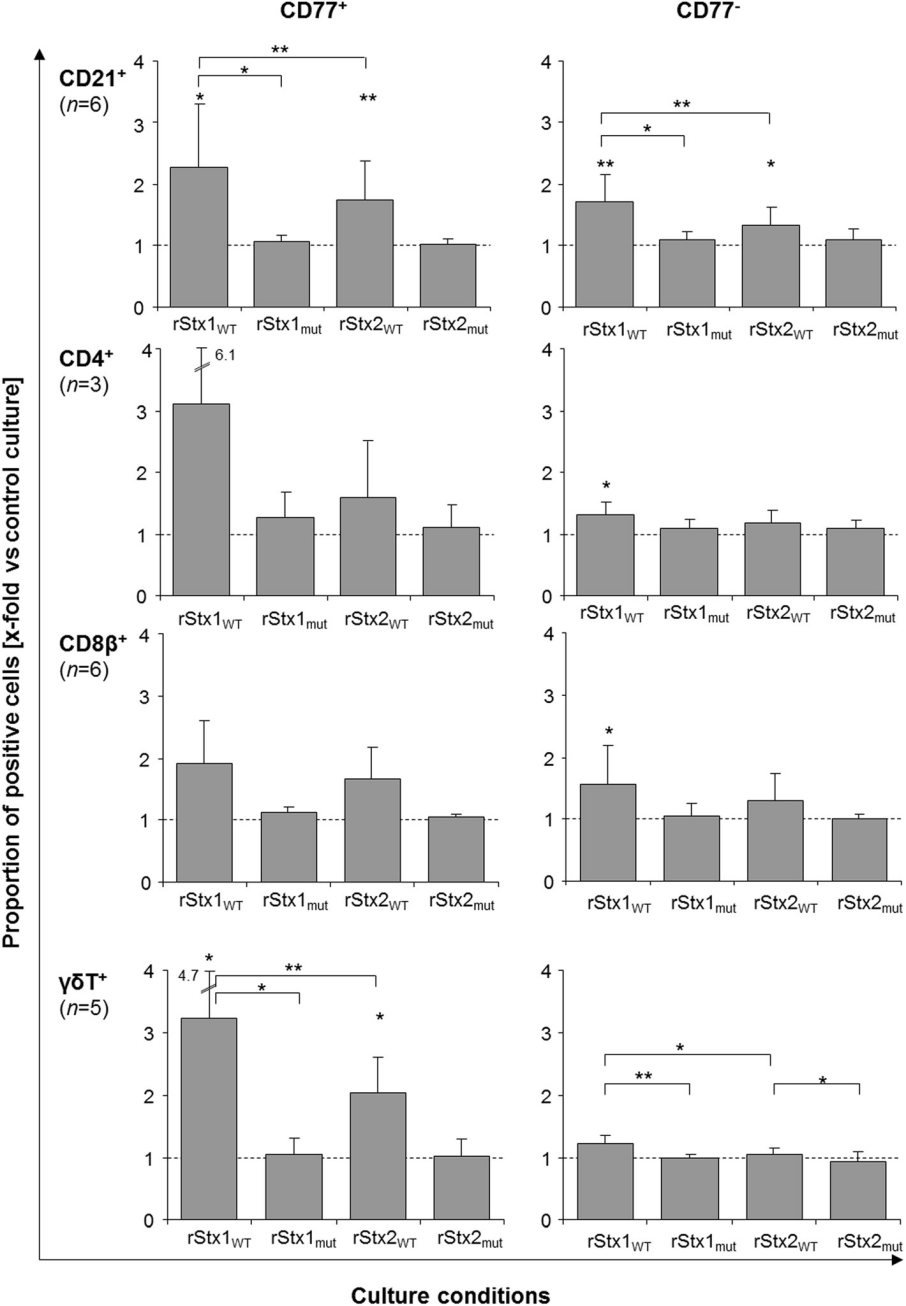
**Late-apoptotic/necrotic cells within PBMC subsets after in vitro challenge with recombinant Shiga toxins and toxoids.** Proportions of 7AAD positive cells expressing (left row) or not expressing CD77 (right row) within PBMC subsets in PHA-P stimulated cultures are shown relative to data obtained from PHA-P stimulated cultures incubated in the presence of the vector control (control cultures; defined as 1.0, indicated by the dashed line). Data is depicted as means ± standard deviations of 3 to 6 repetitive experiments as indicated. Statistical analysis was performed as described in legend to Figure 2. Significance levels were defined as *p* ≤ 0.001 [***], *p* ≤ 0.01 [**], and *p* ≤ 0.05 [*].

Changes in PBMC culture composition induced by rStx_WT_ were partially reflected by alterations in the proportion of non-viable cells. The overall portions of early apoptotic cells within lymphocyte populations were not affected by the presence of rStx_WT_ and rStx_mut_ except a decrease in early apoptotic CD8β^+^ PBMC after incubation with rStx2_mut_ (ANOVA; *p* = 0.009; data not shown). However, wild type toxins induced a significant increase of late apoptotic/necrotic cells within the CD21^+^ population (ANOVA; rStx1_WT_: *p* = 0.004; rStx2_WT_: *p* = 0.036; data not shown) and within the CD4^+^ population (ANOVA; rStx1_WT_: *p* = 0.004; data not shown). Effects became more apparent when analysing the percentage of late apoptotic/necrotic cells in the CD77^+^ and CD77^−^ subsets of the lymphocyte populations separately (Figure 5). Incubation with wild type toxins increased the portion of CD77^+^ late apoptotic/necrotic cells in the CD21^+^, CD4^+^, CD8β^+^, and γδT^+^ subsets even though differences did not always statistically significant levels. CD77^−^ cells within the subsets were less or not affected. Incubation with toxoids neither resulted in proportions of early apoptotic nor of late apoptotic/necrotic cells that were significantly elevated compared to control cultures for any of the subpopulations tested irrespective of CD77 co-expression.

### IL-4 transcription in bovine iIEL upon in vitro challenge with rStx_WT_ and rStx_mut_

**Figure 6:**
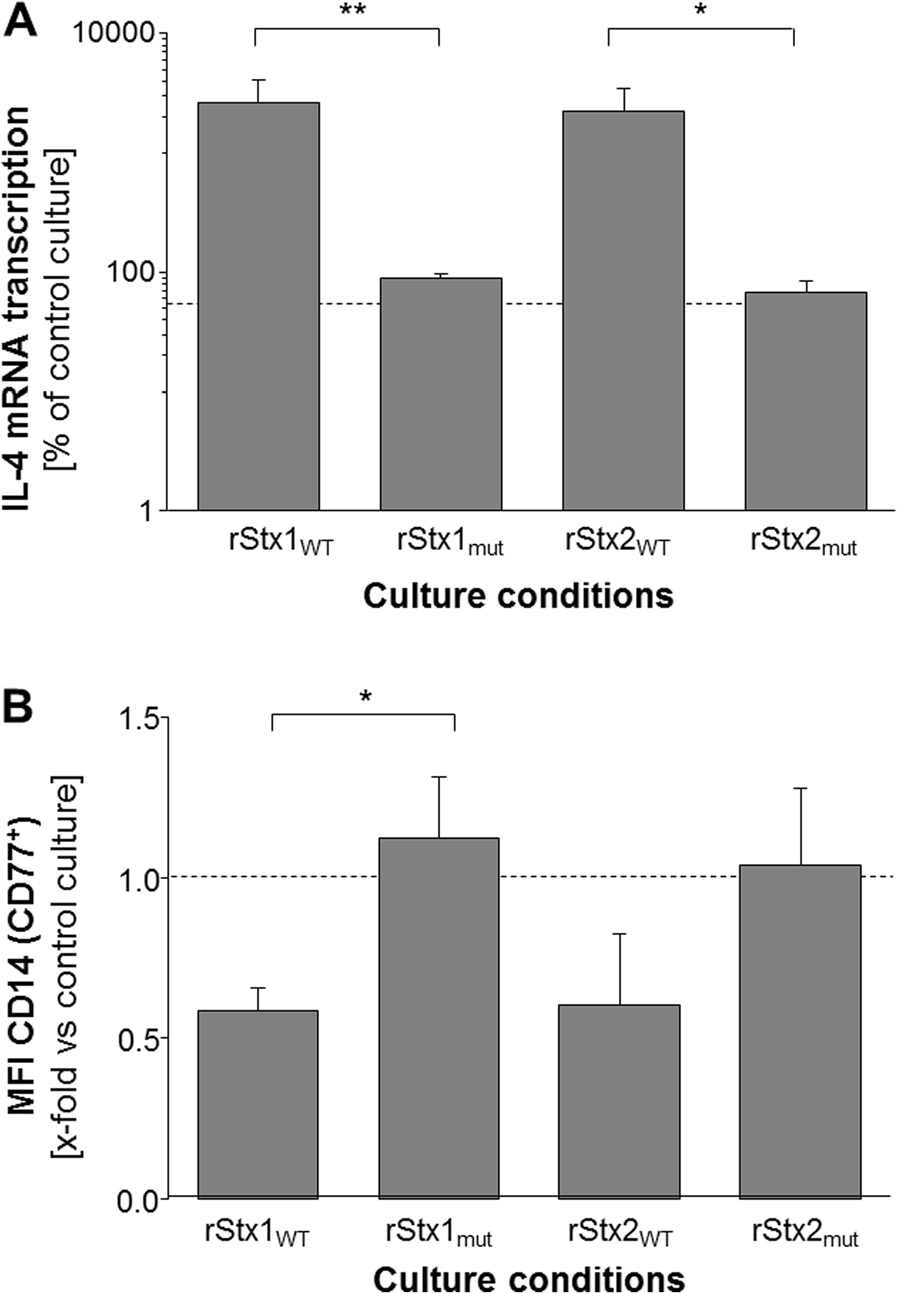
**Gene transcription in bovine iIEL and surface marker expression on bovine MDM incubated for 6 h with rStx**_**WT**_ **or rStx**_**mut**_ **. (A)** Relative amounts of gene transcripts for IL-4 harboured by PHA-P-stimulated (2.5 mg/mL) bovine iIEL normalized to the transcription of the housekeeping gene GAPDH. Vector control cultures were used as reference and set to 100% (dashed line). **(B)** Expression (i.e., mean fluorescence intensity; MFI) of CD14 on bovine CD77^+^ MDM relative to vector control cultures defined as 1.0 (dashed line). Data is depicted as means ± standard deviations from 5 independent experiments each. Statistical analysis was performed as described in legend to Figure 2. Significance levels were defined as *p* ≤ 0.01 [**], and *p* ≤ 0.05 [*].

Ileal intraepithelial lymphocytes (iIEL) are also sensitive to purified wild type Stx1 with a strong induction of IL-4 transcription being the most prominent and reproducible functional implication [9,25]. In corroboration of these findings, incubation of bovine iIEL for 6 h with rStx1_WT_ or rStx2_WT_ both led to a dramatic increase in the amounts of IL-4-specific mRNA (Figure 6A). Again, incubation with comparable amounts of rStx1_mut_ and rStx2_mut_ had no detectable biological effect.

### CD14 expression by bovine MDM upon in vitro challenge with rStx_WT_ and rStx_mut_

Monocyte-derived macrophages (MDM) have recently been discovered as yet another Stx-sensitive cell type in cattle [31]. Incubation with rStx_WT_ or rStx_mut_ for 6 h did not significantly alter the percentage of early apoptotic cells as compared control cultures (data not shown). However, MDM responded to the exposure to rStx1_WT_ or rStx2_WT_ with a clear decrease in the number of CD14 molecules on the cellular surface (as deduced from quantitation of fluorescence intensities (MFI) for the detection of this antigen). The effect was most prominent in the CD77 co-expressing subset of MDM (Figure 6B, data not shown for CD77^−^ MDM). The recombinant toxins had no influence on the number of surface-expressed CD80 and CD86 molecules on CD77^+^ bovine MDM (data not shown). Incubation with comparable amounts of rStx1_mut_ and rStx2_mut_ had no significant effect on CD14, CD80, and CD86 expression by CD77^+^ bovine MDM (Figure 6B; data not shown for CD80 and CD86).

### Recognition of rStx_WT_ and rStx_mut_ by sera from calves naturally exposed to wild type toxins

**Figure 7:**
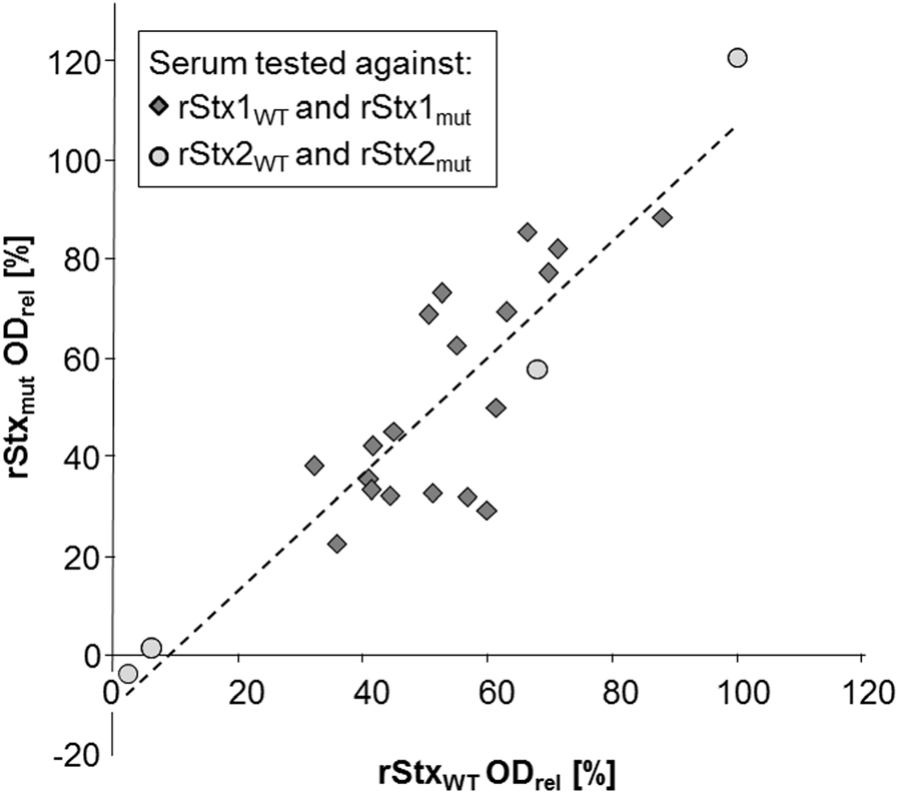
**Antigenicity of recombinant Shiga toxins and toxoids as assessed with calf sera harbouring naturally induced Stx-specific antibodies.** Results obtained with 23 sera in the competitive ELISA are presented. Figure represents OD_rel_ values of defined rStx_WT_ and rStx_mut_ samples, respectively, after pre-incubation with serum (dashed line: trend line [y = 1.179x - 10.702]).

Sera with known specific amounts of Stx antibodies, as defined by VNA and western blotting in a previous study [2], were used to occupy epitopes on rStx_WT_ and rStx_mut_ antigens that were afterwards subjected to the ELISA assay. As the titers of the different sera to Stx1 and Stx2 substantially differed with anti-Stx2 titers being just above the detection limit of the VNA, anti-Stx1 containing sera were pre-diluted to achieve an approx. 50% reduction of the rStx binding to the ELISA plate. Subsequently, pairs of values (rStx_WT_ and rStx_mut_) obtained for the individual sera at a given dilution were analysed by correlation analysis. The competitive ELISA revealed that naturally induced antibodies recognized the corresponding rStx_WT_ and rStx_mut_ equally well (Pearson’s r = 0.886; *p* < 0.001; Figure 7).

### Immunization of calves with rStx1_mut_ and rStx2_mut_

**Figure 8:**
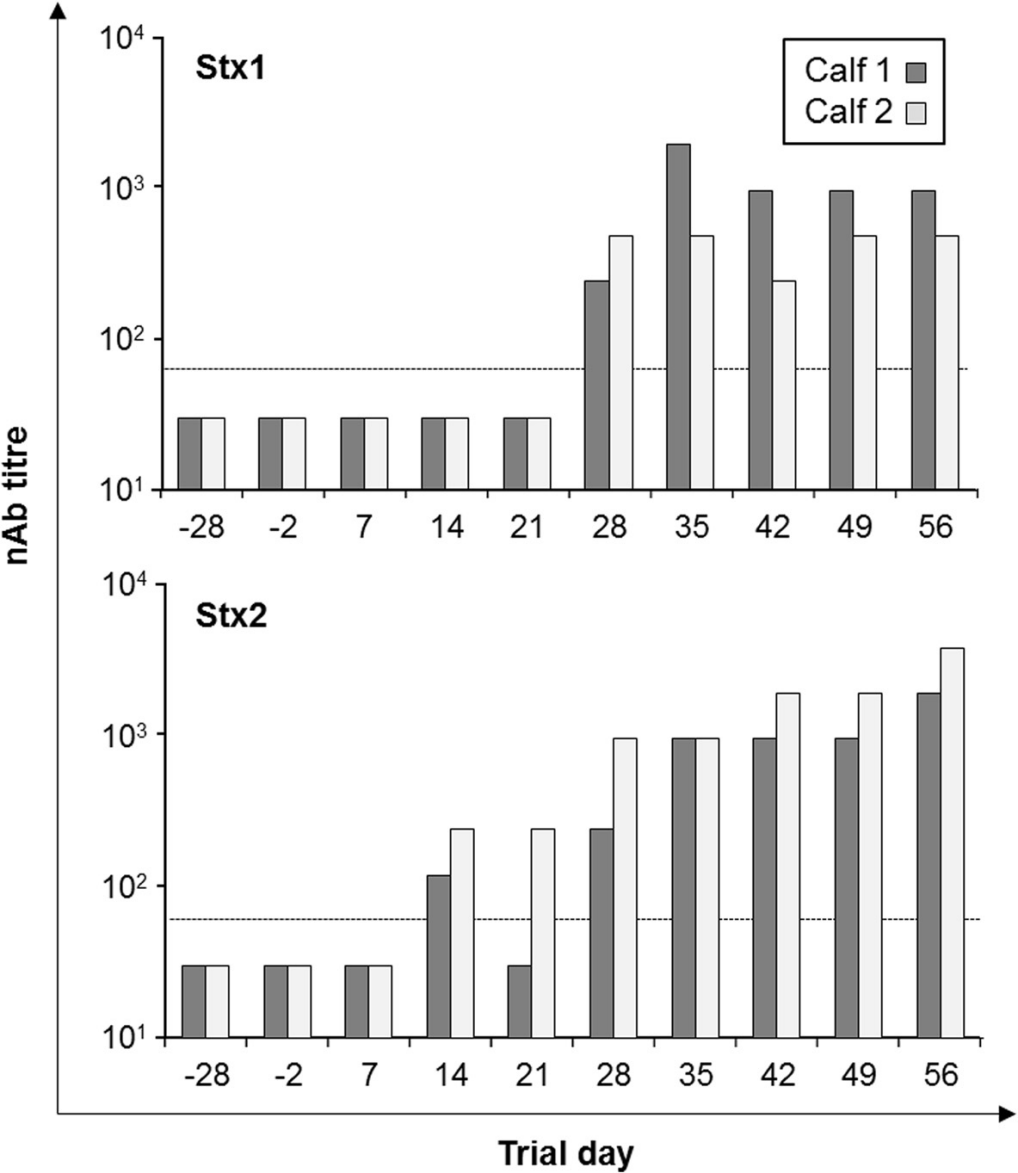
**Detection of Stx1 and Stx2 neutralizing antibodies (nAb) in sera from two calves vaccinated with rStx1**_**mut**_ **and rStx2**_**mut**_ **.** Results from the Vero cell neutralization assay (VNA). Dashed line indicates the detection limit. Calves were vaccinated on days 0 and 21 of the trial. A titre of 30 was attributed to all those samples that gave negative test results in the highest concentration tested.

Until trial day 21, the day of the second immunization with both toxoids, serum samples of both calves tested negative for Stx1 neutralizing antibodies [nAbs] (Figure 8). Beginning one week later, anti-Stx1 nAbs were detectable in both calves. Stx1 nAb titres peaked on trial day 35 and remained on high levels through the end of the trial. Stx2 nAb titres were detectable as early as trial day 14. Titres of calf 1 fell below the detection limit of 60 on trial day 21 and started to rise again beginning on trial day 28. Calf 2 developed a Stx2 nAb titre beginning with trial day 14, one week before the second vaccination. Titre rose constantly until the end of the sampling period.

## Discussion

The immunomodulatory and -suppressive effects of Shiga toxins (Stx) disturb the development of an adaptive immune response against STEC-specific antigens in the course of the initial infection of that far naïve calves [11,12]. In order to develop an effective but biologically safe antigen to vaccinate cattle against the immunologically distinct types 1 and 2 of Stx the objectives of this study were to prove that recombinant Shiga toxoids devoid of the enzymatic activity of the wild type toxins have lost their activity against all types of bovine immune cells identified as potential Stx targets thus far. For functional studies, a concentration of rStx of 200 CD_50_/mL was chosen. Extensive previous studies had shown that purified Stx1 reliably induces maximum modulating effects on bovine immune cells in vitro at this concentration [9]. Concentrations of 2000 CD_50_/mL may induce effects that cannot be fully neutralized by anti-StxB1 and therefore not clearly ascribed to Stx1 even when purified toxin is used [11]. Partially purified (i.e., endotoxin-deprived) preparations were used in the current study causing some depression of the metabolic activity of the robust Vero cells when applied undiluted. As the aim of this study was to provide a broad proof of loss of immunomodulating function of rStx_mut_ in bovines and experiments were exclusively conducted with primary cells availability of which was limited, we refrained from conducting dose–response assays for any of the parameters under study. Consequently, we cannot rule out the possibility that the toxoids may cause adverse effects when applied in significantly higher concentrations. However, efficient induction of a humoral immune response in two calves locally exposed to 1 000 000 CD_50_ equivalents upon vaccination points against such assumptions. Having accomplished this proof-of-principle study presented herein, an extensive experiment is currently under way assessing the immunomodulating, immunogenic, and protective capacity of rStx_mut_-based vaccines under field conditions.

Wild type Stx1 purified from a STEC field isolate blocks the proliferation of bovine peripheral blood T cells, with CD8^+^ T cells in particular, and induces a down-regulation of the Stx receptor CD77 on several lymphocyte subsets without inducing significant cell death by apoptosis or necrosis [11,13]. Results of the present study show that non-purified endotoxin-deprived periplasmic preparations containing recombinant rStx_WT_ induce comparable biological effects in bovine PBMC cultures. Similar to studies with purified Stx1, addition of recombinant rStx_WT_ containing periplasmic preparations did not significantly affect the overall percentage of early or late apoptotic/necrotic cells within major PBMC subsets. In-depth analysis of multicolour flow cytometry data, applied here for the first time, provided evidence, however, that rStx_WT_ treatment has led to an increase in the portion of late apoptotic cells in all lymphocyte populations. Notably, this effect (1) predominantly affected the respective CD77^+^ expressing cells of the subsets and (2) could not be confirmed when analysing for early apoptotic cells. Further studies will be needed to dissect whether this effect can also be induced by purified toxins or is augmented by auxiliary factors present in the periplasmic preparations. Nevertheless, results presented here strongly imply that – despite several reports linking differences in the virulence of EHEC strains for humans to the Stx type or even subtype encoded for by the strains [32] - Stx1 and Stx2 do not differ significantly in their biological activities in bovines, the STEC/EHEC reservoir host, a finding that has direct implications for vaccine development.

Incubation of PBMC with rStx1_mut_ and rStx2_mut_ neither influenced the percentage of lymphocytes expressing CD77^+^ nor the overall subset composition. Toxoids did not induce a down-regulation of CD14 in MDM cultures and did not lead to an up-regulation of IL-4 transcription in iIEL cultures, effects that occurred in the presence of wild type toxins. Even though the molecular mechanism by which Stx induce cell death in a variety of cell lines and primary cells is well understood [33-35], the molecular basis of the immunomodulatory effects of wild type Stx to bovine immune cells is not entirely clear. In most cells, Stx primarily inhibit protein synthesis by acting on the 23S rRNA incorporated in ribosomes [36]. THP-1 cells show an up-regulation of TNF-α upon treatment with Stx1 [37], an effect traced back to the ribotoxic stress response triggered by the enzymatic activity of Stx towards ribosomes. It would also be plausible that cross-linking of CD77 molecules on the cellular surfaces by the multivalent 5B plus 1A structured Stx has initiated cellular responses independent from the enzymatic activity as toxin binding induces apoptosis in sensitive cell lines [14]. We previously showed that incubation of bovine iIEL neither with Stx1 holotoxin nor with purified Stx1B subunit or with anti-CD77 antibody induces IL-4 transcription [9]. By contrast, binding of rStxB1 to CD77 on bovine PBMC induced a holotoxin like activity, e.g., an inhibition of lymphocyte proliferation [14]. The results presented here using genetically modified Stx devoid of verocytotoxic activity as well as lacking any detectable biological activities of rStx1_mut_ and rStx2_mut_ in all in vitro systems applied strongly suggest that the enzymatic activity is essentially required for the immunomodulating effect of Stx in cattle and underscore that the toxoids may represent biologically safe vaccines.

Final prove of biological safety can only come from immunization trials in vivo. Of note, the interferon-α receptor (IFNAR) harbours potential binding sites for CD77 in its extracellular domains, structurally related to CD77 binding sites of StxB subunits [38] raising the possibility that Stx immunization may induce auto-antibodies. The detrimental potential of vaccine-induced auto-antibodies has become dramatically apparent by the occurrence of bovine neonatal panleucocytopenia (BNP). In this clinical entity, prevalent in several European countries in recent years, anti-leukocytic antibodies induced by vaccination of dams are transmitted to their offspring causing severe bleedings and bone marrow depletion [39]. Anti-Stx1 antibodies can frequently be found in adult cattle [2,40] and anti-Stx2 antibodies, although with strikingly lower frequencies and titres, can also be detected. Two calves could be successfully vaccinated by two shots of vaccines containing rStx1_mut_ and rStx2_mut_, and immunization did not exert adverse effects indicative of auto-antibodies.

Antigenicity of rStx_mut_ was evaluated in comparison to rStx_WT_ in a competitive ELISA format using sera obtained from naturally exposed calves with known anti-Stx1 and anti-Stx2 titres [2]. Pre-incubation of the sera with rStx_mut_ and rStx_WT_ equally well reduced binding of the toxins to the capture antibodies. We take this as a strong hint that the structure of important epitopes being the target of a significant portion of naturally induced antibodies are conserved irrespective of the amino acid exchanges in the toxoids introduced by genetic modification.

To be used as vaccine component, inactivated Stx molecules must remain immunogenic. Chemical inactivation of Stx2e by formaldehyde treatment abolishes the cytotoxic effect in vitro but application of the toxoid failed to prevent piglets from developing edema disease upon intravenous challenge with wild type Stx2e [21]. By contrast, inactivation of Stx2e by means of genetic amino acid exchange in the enzymatic cleft of the A subunit resulted in a vaccine able to induce protective antibodies in piglets [41]. Similarly, the survival rate of mice after Stx1 challenge could be raised to 100% when animals had been immunized with mutagenized Stx1 (E167Q, R170L) [23]. It remains unclear whether the poor induction of an anti-Stx response in cattle after natural STEC infection [2] is due to an active immunosuppression by Stx, due to an insufficient antigen exposure by small amounts of toxins produced in vivo or due to poor immunogenicity of the toxins. The latter may result from the structural similarity of StxB subunit with bovine IFNAR and be the consequence of a centrally induced immunological tolerance. Nevertheless, i.m. application to calves of the toxoids generated and characterized in this study led to the induction of substantial anti-Stx1 as well as anti-Stx2 titres, presumably protective in that they at least are able to neutralize the biological activity of Stx holotoxin in vitro. The study design applied here does not allow for concluding on the specificity of the antibodies to each of the toxoids. Kinetics of shedding of Stx1- and Stx2-producing STEC strains as well as kinetics and magnitude of maternal and endogenous anti-Stx antibodies in calves substantially differ [2]. Further studies are worthwhile to separately optimize the immunogenic capacity of the two toxoids and to assess their relative protective efficacy, e.g., by modifications of the vaccine formulation and application scheme.

The STEC/EHEC pathovar consists of a plethora of different *E. coli* strains varying in serotype and virulence gene pattern. By definition, Stx’s are the only virulence factors harboured by all STEC strains. Up to now, success of attempts to vaccinate cattle was mostly restricted to single subpopulations of STEC, e.g. strains positive for O157 [42], harbouring the genes for Tir (translocated intimin receptor) [42], for adhesion factor intimin [43], Esp’s (*E. coli* secreted proteins) A and B [44,45] or flagellin H7 [46]. Stx rather act as immunomodulating agents during bovine STEC infections [8-12] by affecting the early phases of immune activation than by depressing an established immunity [13,14]. Consequently, Stx may principally be effective upon first STEC infection of hitherto immunologically naïve animals at the time they first encounter STEC antigens. In the absence of Stx, animals may be able to mount an efficient adaptive immune response with the potential to prevent persistent STEC colonization of the intestinal mucosa. However, Stx always co-occurs with STEC antigens in spatial and temporal terms during infection. In this particular situation, Stx apparently hinders calves from properly responding, creating an immunologically privileged niche and thereby paving the way for persistent colonization. Application of Stx toxoid-based vaccines may enable calves to actively mount a primary immune response to antigens other than Stx that are harboured by STEC strains circulating in the respective cohort. In case future studies show that this does not suffice, subsequent application of aforementioned vaccines as booster shall be evaluated as to their ability to eventually induce a robust anti-STEC adaptive immune response mitigating long-term STEC shedding by cattle.

## References

[1] Caprioli A, Morabito S, Brugere H, Oswald E (2005). Enterohaemorrhagic *Escherichia coli*: emerging issues on virulence and modes of transmission. Vet Res.

[2] Fröhlich J, Baljer G, Menge C (2009). Maternally and naturally acquired antibodies to Shiga toxins in a cohort of calves shedding Shiga-toxigenic *Escherichia coli*. Appl Environ Microbiol.

[3] Naylor SW, Gally DL, Low JC (2005). Enterohaemorrhagic *E. coli* in veterinary medicine. Int J Med Microbiol.

[4] Geue L, Segura-Alvarez M, Conraths FJ, Kuczius T, Bockemühl J, Karch H, Gallien P (2002). A long-term study on the prevalence of shiga toxin-producing *Escherichia coli* (STEC) on four German cattle farms. Epidemiol Infect.

[5] Vande Walle K, Vanrompay D, Cox E (2013). Bovine innate and adaptive immune responses against *Escherichia coli* O157:H7 and vaccination strategies to reduce faecal shedding in ruminants. Vet Immunol Immunopathol.

[6] Snedeker KG, Campbell M, Sargeant JM (2012). A systematic review of vaccinations to reduce the shedding of *Escherichia coli* O157 in the faeces of domestic ruminants. Zoonoses Public Health.

[7] Mahajan A, Currie CG, Mackie S, Tree J, McAteer S, McKendrick I, McNeilly TN, Roe A, La Ragione RM, Woodward MJ, Gally DL, Smith DG (2009). An investigation of the expression and adhesin function of H7 flagella in the interaction of *Escherichia coli* O157:H7 with bovine intestinal epithelium. Cell Microbiol.

[8] Stamm I, Mohr M, Bridger PS, Schröpfer E, König M, Stoffregen WC, Dean-Nystrom EA, Baljer G, Menge C (2008). Epithelial and mesenchymal cells in the bovine colonic mucosa differ in their responsiveness to *Escherichia coli* Shiga toxin 1. Infect Immun.

[9] Moussay E, Stamm I, Taubert A, Baljer G, Menge C (2006). *Escherichia coli* Shiga toxin 1 enhances *il-4* transcripts in bovine ileal intraepithelial lymphocytes. Vet Immunol Immunopathol.

[10] Menge C, Stamm I, Van Diemen PM, Sopp P, Baljer G, Wallis TS, Stevens MP (2004). Phenotypic and functional characterization of intraepithelial lymphocytes in a bovine ligated intestinal loop model of enterohaemorrhagic *Escherichia coli* infection. J Med Microbiol.

[11] Menge C, Wieler LH, Schlapp T, Baljer G (1999). Shiga toxin 1 from *Escherichia coli* blocks activation and proliferation of bovine lymphocyte subpopulations in vitro. Infect Immun.

[12] Hoffman MA, Menge C, Casey TA, Laegreid W, Bosworth BT, Dean-Nystrom EA (2006). Bovine immune response to shiga-toxigenic *Escherichia coli* O157:H7. Clin Vaccine Immunol.

[13] Menge C, Stamm I, Wuhrer M, Geyer R, Wieler LH, Baljer G (2001). Globotriaosylceramide (Gb_3_/CD77) is synthesized and surface expressed by bovine lymphocytes upon activation in vitro. Vet Immunol Immunopathol.

[14] Stamm I, Wuhrer M, Geyer R, Baljer G, Menge C (2002). Bovine lymphocytes express functional receptors for *Escherichia coli* Shiga toxin 1. Microb Pathog.

[15] Kuribayashi T, Seita T, Fukuyama M, Furuhata K, Honda M, Matsumoto M, Seguchi H, Yamamoto S (2006). Neutralizing activity of bovine colostral antibody against verotoxin derived from enterohemorrhagic *Escherichia coli* O157:H7 in mice. J Infect Chemother.

[16] Kuribayashi T, Seita T, Matsumoto M, Furuhata K, Tagata K, Yamamoto S (2009). Bovine colostral antibody against verotoxin 2 derived from *Escherichia coli* O157:H7: resistance to proteases and effects in beagle dogs. Comp Med.

[17] Johnson RP, Cray WC, Johnson ST (1996). Serum antibody responses of cattle following experimental infection with *Escherichia coli* O157:H7. Infect Immun.

[18] MacLeod DL, Gyles CL (1991). Immunization of pigs with a purified Shiga-like toxin II variant toxoid. Vet Microbiol.

[19] Hovde CJ, Calderwood SB, Mekalanos JJ, Collier RJ (1988). Evidence that glutamic acid 167 is an active-site residue of Shiga-like toxin I. Proc Natl Acad Sci U S A.

[20] Yamasaki S, Furutani M, Ito K, Igarashi K, Nishibuchi M, Takeda Y (1991). Importance of arginine at position 170 of the A subunit of Vero toxin 1 produced by enterohemorrhagic *Escherichia coli* for toxin activity. Microb Pathog.

[21] Makino S, Watarai M, Tabuchi H, Shirahata T, Furuoka H, Kobayashi Y, Takeda Y (2001). Genetically modified Shiga toxin 2e (Stx2e) producing *Escherichia coli* is a vaccine candidate for porcine edema disease. Microb Pathog.

[22] Ohmura-Hoshino M, Yamamoto M, Yuki Y, Takeda Y, Kiyono H (2004). Non-toxic Stx derivatives from *Escherichia coli* possess adjuvant activity for mucosal immunity. Vaccine.

[23] Ishikawa S, Kawahara K, Kagami Y, Isshiki Y, Kaneko A, Matsui H, Okada N, Danbara H (2003). Protection against Shiga toxin 1 challenge by immunization of mice with purified mutant Shiga toxin 1. Infect Immun.

[24] Gentry MK, Dalrymple JM (1980). Quantitative microtiter cytotoxicity assay for *Shigella* toxin. J Clin Microbiol.

[25] Menge C, Blessenohl M, Eisenberg T, Stamm I, Baljer G (2004). Bovine ileal intraepithelial lymphocytes represent target cells for Shiga toxin 1 from *Escherichia coli*. Infect Immun.

[26] Adler H, Peterhans E, Jungi TW (1994). Generation and functional characterization of bovine bone marrow-derived macrophages. Vet Immunol Immunopathol.

[27] Werling D, Howard CJ, Niederer E, Straub OC, Saalmüller A, Langhans W (1998). Analysis of the phenotype and phagocytic activity of monocytes/macrophages from cattle infected with the bovine leukaemia virus. Vet Immunol Immunopathol.

[28] Barth S, Duncker S, Hempe J, Breves G, Baljer G, Bauerfeind R (2009). *Escherichia coli* Nissle 1917 for probiotic use in piglets: evidence for intestinal colonization. J Appl Microbiol.

[29] Nguyen TV, Le Van P, Le Huy C, Gia KN, Weintraub A (2005). Detection and characterization of diarrheagenic *Escherichia coli* from young children in Hanoi, Vietnam. J Clin Microbiol.

[30] Menge C, Stamm I, Blessenohl M, Wieler LH, Baljer G (2003). Verotoxin 1 from *Escherichia coli* affects Gb_3_/CD77^+^ bovine lymphocytes independent of interleukin-2, tumor necrosis factor-alpha, and interferon-alpha. Exp Biol Med.

[31] Loos D (2012) Untersuchungen zum Einfluss von Shigatoxin auf Makrophagen und dendritische Zellen des Rindes. Doctoral thesis, Justus Liebig University Gießen, Institute of Hygiene and Infectious Diseases of Animals.

[32] Friedrich AW, Bielaszewska M, Zhang WL, Pulz M, Kuczius T, Ammon A, Karch H (2002). *Escherichia coli* harboring Shiga toxin 2 gene variants: frequency and association with clinical symptoms. J Infect Dis.

[33] O’Loughlin EV, Robins-Browne RM (2001). Effect of Shiga toxin and Shiga-like toxins on eukaryotic cells. Microbes Infect.

[34] Karmali MA (2004). Infection by Shiga toxin-producing *Escherichia coli*: an overview. Mol Biotechnol.

[35] Foster GH, Tesh VL (2002). Shiga toxin 1-induced activation of c-Jun NH(2)-terminal kinase and p38 in the human monocytic cell line THP-1: possible involvement in the production of TNF-alpha. J Leukoc Biol.

[36] Obrig TG, Moran TP, Brown JE (1987). The mode of action of Shiga toxin on peptide elongation of eukaryotic protein synthesis. Biochem J.

[37] Harrison LM, van Haaften WC, Tesh VL (2004). Regulation of proinflammatory cytokine expression by Shiga toxin 1 and/or lipopolysaccharides in the human monocytic cell line THP-1. Infect Immun.

[38] Maloney MD, Binnington-Boyd B, Lingwood CA (1999). Globotriaosyl ceramide modulates interferon-alpha-induced growth inhibition and CD19 expression in Burkitt’s lymphoma cells. Glycoconj J.

[39] Bridger PS, Bauerfeind R, Wenzel L, Bauer N, Menge C, Thiel HJ, Reinacher M, Doll K (2011). Detection of colostrum-derived alloantibodies in calves with bovine neonatal pancytopenia. Vet Immunol Immunopathol.

[40] Pirro F, Wieler LH, Failing K, Bauerfeind R, Baljer G (1995). Neutralizing antibodies against Shiga-like toxins from *Escherichia coli* in colostra and sera of cattle. Vet Microbiol.

[41] Gordon VM, Whipp SC, Moon HW, O’Brien AD, Samuel JE (1992). An enzymatic mutant of Shiga-like toxin II variant is a vaccine candidate for edema disease of swine. Infect Immun.

[42] Potter AA, Klashinsky S, Li Y, Frey E, Townsend H, Rogan D, Erickson G, Hinkley S, Klopfenstein T, Moxley RA, Smith DR, Finlay BB (2004). Decreased shedding of *Escherichia coli* O157:H7 by cattle following vaccination with type III secreted proteins. Vaccine.

[43] van Diemen PM, Dziva F, Abu-Median A, Wallis TS, van den Bosch H, Dougan G, Chanter N, Frankel G, Stevens MP (2007). Subunit vaccines based on intimin and Efa-1 polypeptides induce humoral immunity in cattle but do not protect against intestinal colonisation by enterohaemorrhagic *Escherichia coli* O157:H7 or O26:H. Vet Immunol Immunopathol.

[44] Dziva F, Vlisidou I, Crepin VF, Wallis TS, Frankel G, Stevens MP (2007). Vaccination of calves with EspA, a key colonisation factor of *Escherichia coli* O157:H7, induces antigen-specific humoral responses but does not confer protection against intestinal colonisation. Vet Microbiol.

[45] Bretschneider G, Berberov EM, Moxley RA (2007). Isotype-specific antibody responses against *Escherichia coli* O157:H7 locus of enterocyte effacement proteins in adult beef cattle following experimental infection. Vet Immunol Immunopathol.

[46] McNeilly TN, Naylor SW, Mahajan A, Mitchell MC, McAteer S, Deane D, Smith DG, Low JC, Gally DL, Huntley JF (2008). *Escherichia coli* O157:H7 colonization in cattle following systemic and mucosal immunization with purified H7 flagellin. Infect Immun.

